# The Environmental and Global Impact of Pharmacogenomics: Advancing Green Pharmacy Toward Sustainable and Inclusive Precision Medicine

**DOI:** 10.3390/jpm16040183

**Published:** 2026-03-27

**Authors:** Pálma Porrogi

**Affiliations:** Faculty of Health and Sport Sciences, Széchenyi István University, 9026 Gyor, Hungary; porrogi.palma@ga.sze.hu

**Keywords:** pharmacogenomics, Cytochrome P450, green pharmacy, healthcare sustainability, precision medicine, pharmaceutical waste, genetic admixture, clinical decision support

## Abstract

Traditional one size fits all pharmacotherapy often yields suboptimal clinical outcomes, preventable adverse drug reactions (ADRs), and significant drug waste, imposing substantial economic and ecological burdens on healthcare systems. This review evaluates the transformative potential of pharmacogenomics (PGx) testing, particularly cytochrome *P450* (CYP) gene variants, as a foundation for an ecosystem-centric accountability framework for green pharmacy and links human metabolic variability to specific environmental outcomes. Personalized CYP profiling is shown to minimize the environmental release of unused drugs and potentially ecotoxic metabolites into aquatic ecosystems, in contrast to standard uniform drug use approaches. The limitations of ethnicity-based dosing models, which rely on population genetic variation, are examined in the context of increasing global genetic admixture. It is argued that individual genetic profiling, conceptualized as a PGx-Green Passport, provides a reliable safety standard that accounts for individual differences, thereby enhancing efficiency and well-being in a globalized society. By integrating clinical data, including real-world evidence on hospital utilization, with sustainability frameworks, this review demonstrates that PGx-guided therapy is not only a tool for clinical efficiency but also a fundamental requirement for systematically achieving environmentally sustainable healthcare.

## 1. Introduction

**Figure 1:**
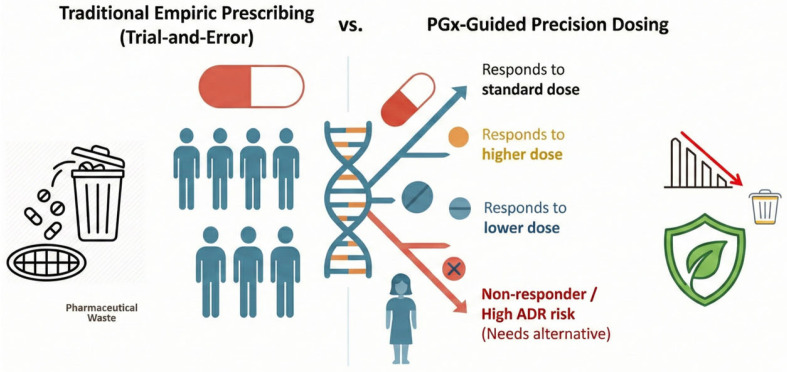
The comparison of empiric and precision pharmacotherapy. The (**left**) panel illustrates the traditional one-size-fits-all approach, in which uniform dosing yields variable responses, higher rates of therapeutic failure and adverse reactions, and significant pharmaceutical waste and environmental burden (indicated by the rising graph). The (**right**) panel demonstrates the PGx-guided model, in which individual DNA profiling guides precise drug selection and dosing. This targeted approach maximizes clinical efficacy while sharply reducing unused medication waste and the resulting ecological footprint (as indicated by the falling graph and sustainability icon).

Precision medicine represents a paradigm shift in healthcare, moving beyond the traditional “one size fits all” model toward a personalized approach that accounts for individual variability in genes, environment, and lifestyle [1,2,3]. This innovative shift aims to optimize disease prevention and treatment by moving away from medical recommendations based on the average patient and on a single dose (Figure 1). At the heart of this transition is pharmacogenomics (PGx)—the DNA-based foundation of precision medicine that identifies how genetic variations influence the metabolic activity of individual drugs.

The lack of global clinical use of PGx has resulted in a significant reduction in serious adverse events and toxicity in patients, even though genetically based phenotype prediction could be scientifically feasible, thereby reducing the ecological and economic burden on the health system. Although the scientific evidence supporting the use of genomic data in drug therapy is solid, several barriers and constraints hinder its widespread adoption and implementation in routine clinical practice [4,5,6,7]. Significant barriers include the lack of specialized training for healthcare professionals (physicians and pharmacists), limited access to genetic testing, and the complexity of implementing the Clinical Pharmacogenetics Implementation Consortium (CPIC) guidelines [8,9,10,11,12]. Furthermore, translating genomic data into practical clinical decisions is often hampered by the lack of sophisticated clinical decision support (CDS) systems, which are essential for deriving accurate therapeutic recommendations from vast PGx datasets.

Despite the global adoption of PGx, its integration into routine clinical practice remains hampered by a lack of standardized education and by the complexity of interpreting genomic data and applying it in practice [5,13,14,15]. Despite the scientific knowledge, the potential for application remains a gap between theory and clinical application. The main goal of the precision medicine approach is to bridge this gap. In this case, it requires a complex, multi-point intervention, education, openness, and a shift in perspective, especially when dealing with the global environmental problems of the earth. After all, one of the main pillars of environmental protection is the conscious, justified use of medicines. This can be best achieved by optimizing the dosing of PGx-based drugs. To overcome barriers to practical implementation, the CPIC was established to provide expert-reviewed, real-world, evidence-based guidelines that facilitate the practical interpretation of genetic laboratory test results and the formulation of pharmacological prescribing decisions [1,3]. These guidelines are essential for minimizing adverse drug reactions (ADRs) and optimizing therapeutic efficacy through standardized genotype-phenotype interpretation [16]. However, let us not forget that science has advanced to the point that we also know that phenotypic metabolic activity is not a direct consequence of genotype, as many individual molecular interactions (inhibition, induction, silencing) or acquired epigenetic patterns at the DNA level can significantly influence the phenotypic manifestation of the genotypic pattern. But for now, our goal is to at least provide knowledge of acquired gene variants as a basic starting point for drug therapy.

Recent updates further emphasize the role of PGx in promoting a sustainable healthcare ecosystem. For example, updated guidelines for CYP2D6-guided opioid therapy and beta-blocker dosing, as well as CYP2C19-guided treatment of proton pump inhibitors, demonstrate how precision dosing can significantly reduce the environmental footprint of drugs [4,9,10,17,18]. By preventing the prescription of ineffective drugs, these evidence-based strategies will reduce systemic drug waste and the chemical burden on global wastewater systems, stemming from both direct drug degradation products and primary and secondary toxic metabolites [19,20]. Thus, the implementation of CPIC standardized protocols is not only a clinical necessity for patient safety, but also a strict strategic requirement for green, environmentally conscious pharmacy and long-term health sustainability [12,19,21,22,23].

A novel and critical dimension of this review is the evaluation of PGx through the lens of sustainability. The World Health Organization (WHO) defines a sustainable health system as one that improves or restores health while minimizing adverse environmental impacts [24]. Environmental sustainability in healthcare emphasizes value-based care and the use of personalized medicine to optimize resource allocation and reduce costs (Figure 1). We hypothesize that CYP-based personalized protocols are a fundamental bridge to effective drug treatment for a growing global population [3,25]. By mitigating the widespread issue of pharmaceutical waste and preventing the prescribing of ineffective medications, PGx directly aligns with the WHO’s mission of professionalism, efficiency, and sustainability [5,26,27]. A novel and critical dimension of this review is the assessment of PGx from a sustainability perspective. The WHO defines a sustainable health system as one that improves or restores health while minimizing adverse environmental impacts [24,26,28]. Environmental sustainability in health emphasizes value-based care and the use of personalized medicine to optimize resource allocation and reduce costs. We argue that CYP-based personalized protocols provide an essential bridge to effective medication management for a growing global population [3,29]. By alleviating the widespread problem of medication waste and preventing the prescribing and use of ineffective medications, PGx-based drug therapy aligns with the WHO’s mission to provide professional, efficient, and ecologically sustainable health care [24]. While Standard PGx focuses on the patient-centric axis of efficacy and safety, Green PGx introduces a multi-layered accountability model. In this framework, genetic data is used to predict and mitigate ecological ADRs, unintentional environmental consequences arising from altered drug excretion patterns in specific genotypes [30].

## 2. Research Gap and Strategic Objectives

Despite the clinical benefits and the push for widespread routine use of CYP-based PGx drug therapy, there remains a significant research gap in understanding its systemic impact on healthcare sustainability and its role in a rapidly globalizing society. The current literature often relies on ethnicity as a proxy for genetic variability, and this remains a determinant in clinical applications. For example, routine genotyping of a child in oncohematology is not performed because the given gene variant is rare in the Caucasian population (e.g., for CYP isoenzymes and other major gene variants involved in drug metabolism) [31,32,33]. But most of the time, these can be measured in a sample, for example, in a multigene oncology panel. So we are still at a point where the population distribution of most CYP isoenzyme variants is well known, but the increasing global population mixing and genetic admixture are completely ignored. Only experience-based prediction remains. This approach, and the lack of application of knowledge and recommendations, render traditional ethnic-based drug dosing models obsolete, thereby compromising patient well-being, increasing adverse events, and significantly burdening the healthcare system. I propose that individual genetic profiling is a fundamental tool for understanding metabolic tendencies. Human cellular processes can be understood within a broader multi-omics framework where genetic identity is a key component. Combining genetic markers with dynamic metabolomics data is crucial to capture real-time metabolic activity, thereby extending the usefulness of PGx data beyond clinical safety into a measurable environmental risk framework [34]. This genetically based, multi-layered approach will enable more accurate and efficient clinical decisions, optimize healthcare resource allocation, and greatly reduce pharmaceutical waste [35]. This will reduce both the adverse effects (liver damage, hepatotoxicity, acute pancreatitis) that patients experience due to the use of inappropriate types and doses of drugs, as well as the amount of harmful, toxic substances released into the environment. Unfortunately, the intersection of PGx and the green pharmacy movement has remained largely undeveloped [36]. A critical, yet overlooked, ecological advantage is that a single, comprehensive, multigene PGx panel is inherently more sustainable than repeated conventional laboratory monitoring without baseline genetic information. Traditional trial-and-error approaches and frequent biochemical measurements require continuous inventory management of reagents, plastic consumables, and energy-intensive supplies, leading to larger volumes of potentially ecotoxic pharmaceutical and laboratory waste, as well as greater human resource use, unnecessarily burdening healthcare personnel. This review aims to bridge these gaps by introducing an Eco-gene Matrix and an Eco-score system that assigns specific ecological indices to genetic variants. By demonstrating that preventive PGx testing is not only a tool for patient safety but also an essential strategy for reducing the environmental footprint of modern medicine, we provide a roadmap for the integrated assessment of clinical and ecological outcomes [37]. Finally, focusing on sustainability while effectively addressing knowledge gaps and future challenges related to new, currently unknown gene variants, variant combinations, and their frequencies in ethnic genetic mixtures can be achieved through a genotype-based, so-called genotype-evidence-based drug therapy.

The review specifically addresses the following objectives:

1. PGx is a pillar of green pharmacy, complementing patient safety with ecosystem accountability. Metabolic optimization reduces pharmaceutical ecotoxicity.
2. The PGx Green Passport is proposed as a lifelong standard containing genotype data, which can trigger ethnic dosing and enable precise therapy for different populations.
3. This new methodology assigns ecological indices to genetic data using the Eco-gene Matrix and the Eco Score. This links metabolic profiles to environmental impact and guides sustainable drug selection.

## 3. Methods

This article uses a narrative review to synthesize current knowledge on the role of PGx in advancing green pharmacy and sustainable, targeted therapies. The goal was to provide a comprehensive yet concise review that integrates clinical molecular biology with environmental awareness. I conducted a thorough, non-systematic literature search in multiple electronic databases, including PubMed, Scopus, Web of Science, and Google Scholar. The search included peer-reviewed articles, clinical guidelines (e.g., CPIC, DPWG), and recent systematic reviews published up to early 2026 [4]. The literature search strategy was applied to the following keywords and their combinations: Pharmacogenomics or PGx, Green pharmacy, Sustainable healthcare, SNPs, Cytochrome P450 or CYP variability, hybridization or genetic admixture in drug response, Pharmaceutical waste, and Ecotoxicity.

The articles were prioritized and selected based on their relevance to drug metabolism mediated by CYP enzymes, their clinical utility in preventing adverse drug reactions, and their contribution to reducing the environmental footprint of pharmacotherapy. Special emphasis was placed on translating scientific findings into practical clinical and environmental strategies.

## 4. Results

### 4.1. Pharmaceutical Waste and Ecotoxicity: The Environmental Cost of Clinical Inefficiency

The environmental footprint of modern drug therapy has reached a critical threshold, where pharmaceuticals are now recognized as persistent anthropogenic micropollutants of global concern [22]. A significant portion of this ecological burden results directly from the inefficiency of traditional drug selection and dosing. When drugs are clinically ineffective, toxic, or induce other undesirable side effects or organ dysfunctions due to the metabolic activity determined by the individual genotype, significant amounts of unused pharmaceutical waste often result, which are often inadequately stored and disposed of. Furthermore, even after drugs enter the body, they are often not completely metabolized; instead, they are excreted in human urine and feces as active compounds or potentially toxic metabolites, which then enter wastewater systems [20]. Most current wastewater treatment plants are not designed to remove, or even degrade, these complex organic molecules, which continuously pollute aquatic ecosystems [20,38]. This chronic exposure poses serious ecotoxicological risks, including endocrine disruption and bioaccumulation in non-target organisms, ultimately transforming the goal of protecting human health into a driver of unintentional environmental damage [6,19,20,39].

While traditional pharmacovigilance predominantly emphasizes patient safety, Green PGx expands this responsibility to encompass the patient-out phase, addressing the downstream environmental implications of pharmacotherapy [40].

The eco-genetic matrix is a conceptual framework for quantifying the influence of individual genetic variability on the quantity and quality of xenobiotics released into the environment. Thus, Green PGx serves a dual function: first, it characterizes interindividual differences in drug-metabolizing enzymes, and second, it highlights the potential for environmentally relevant pharmaceutical pollution. Accordingly, tailoring pharmacotherapy based on Green PGx principles can yield not only improved therapeutic efficacy and reduced adverse drug reactions at the individual level, but also a diminished environmental burden of toxic drug metabolites. Furthermore, by minimizing the administration of ineffective medications, the associated generation of single-use plastic waste is also reduced, thereby contributing to a lower overall ecological footprint.

Based on the specific metabolic mechanisms and potential hazards of the substance detailed in the eco-genetic matrix, we propose assigning a uniform metric to the genetic data to quantify the ecological burden from the phenotypic manifestation of the variant (Table 1). The Eco-score could be a composite, indicative index that integrates the ecotoxicological efficacy and environmental persistence of a drug with the increase in drug excretion driven by genotype [38,39]. It represents the cumulative environmental risk posed by a drug-patient genetic interaction.

The CYP3A4*22 allele is one of the critical allele variants in the PGx-Green Passport. Since synthetic estrogens, such as ethinyl estradiol, are active at nanogram/liter levels, up to a 20–30% increase in excretion due to poor metabolism can be observed in local wastewater. This makes testing for the CYP3A4 enzyme a primary environmental intervention (Table 1).

### 4.2. Obsolescence of the Evidence-Based Model

Relying on the empirical one-dose approach to prescribing inherently leads to systemic drug waste, significant side effects, and ineffective treatment [41,42]. A prime example of this is the use of codeine in CYP2D6 poor metabolizers (PMs) [21,43]. In these individuals, the drug has no effect because an enzyme deficiency prevents the prodrug from being converted into active morphine. As a result, the tablets provide no therapeutic benefit and become pharmaceutical waste [35]. This not only leads to clinical failure and environmental contamination, but also creates unnecessary economic costs.

Currently, switching drugs and doses requires recognition of treatment inefficiency. Similarly, genetic variability in *CYP2C9* often leads to inaccurate warfarin dosing [4,9]. Treatment of subsequent overdose requires immediate drug withdrawal, which forces the destruction of expensive, partially used packages and increases the chemical burden on the healthcare system.

The clinically proven link between PGx guidance and the efficacy and success of better first-line drugs directly supports the goal of reducing unused drug inventory. This link demonstrates that clinical effectiveness and environmental sustainability are inextricably linked. Preventive measurement of CYP enzyme variability prior to drug selection is critical to prevent a negative chain reaction: a sequence that ranges from therapeutic failure to repeat hospitalization due to toxic events, resulting in significant financial loss and ecological waste [16,19,44,45].

By translating and applying genetic knowledge to guide clinical decisions, healthcare providers can achieve precise drug matching and immediate impact. Thus, PGx is not just a specialized diagnostic tool, but a fundamental strategy to align patient-centered biology with global sustainability goals.

The traditional one-size-fits-all prescribing model is not only inefficient but also a significant source of iatrogenic injury and environmental contamination [2]. If genetic variability is ignored, the resulting trial-and-error process leads to preventable and predictable ADRs [16,26,45,46].

### 4.3. Typical Examples of Adverse Reactions Caused by CYP Polymorphisms

Effect on clopidogrel: Patients carrying loss-of-function alleles of the primary metabolizing enzyme (CYP2C19*2, *3) of the drug are unable to convert the prodrug clopidogrel to its active metabolite [47,48,49]. In East Asian populations, where the frequency of these alleles can reach 30–50%, standard doses significantly increase the risk of serious adverse cardiovascular events.

The commonly used painkillers codeine and tramadol are metabolized by the CYP2D6 isoenzyme [50,51,52]. Ultra-rapid metabolizers (UM), which are common in North African and Middle Eastern populations (up to 10–28%), can experience life-threatening opioid toxicity even at standard doses, while PM (7–10% of Caucasians) do not produce analgesic effects, leading to ineffective treatment and wasted medication [32,50,53]. A third common and well-known example is SSRIs, which are metabolized by multiple CYP isoenzymes (CYP2C19/CYP2D6), thereby contributing to the phenotypic effects caused by gene variants [33,48,54]. Up to 40–50% of patients may fail to achieve the desired effect in their first antidepressant trial due to rapid metabolic activity (UM status) or increased side effects in PM, where the drug and its metabolite circulate in higher concentrations in the blood plasma and for longer. This standard-dose approach with inadequate efficacy often results in the patient being prescribed additional medications to treat the initial side effects, which cascades, sometimes exponentially, the chemical and toxicological burden on the patient and the environment.

Pharmacon dose optimization is a precision medicine process that integrates patient-specific pharmacokinetic, pharmacodynamic, and PGx variability to determine the minimum effective dose of a drug that maximizes clinical response within the therapeutic window while minimizing substrate toxicity and environmental drug burden [40,55]. PGx-guided drug selection significantly reduces the rate of rehospitalizations within 30 days that are specifically attributable to ineffective treatment. By accurately categorizing these rehospitalizations, attributing them to drug-related causes, and applying PGx-based pharmacon dose optimization based on this categorization, we improve our efficiency and eco-friendly, sustainability indicators [51].

### 4.4. Comprehensive Multigene PGx Panels

In addition to single-gene testing, the introduction of multigene PGx panels provides a basis for realizing the green pharmacy [19,56,57]. These panels use next-generation sequencing (NGS) or high-throughput RT-PCR for genotyping and transcriptomic metabolic mapping [6]. Most multigene panels are now available by disease type, focusing on oncology, psychiatry, cardiology, and other disease groups [46,57]. Using such a panel, up to hundreds of gene variants and transcriptomic patterns can be determined from a single sample [6,33,45,51,57]. For example the following tests are recommended: AmpliChip CYP450 test [58,59], ClinPGx-driven NGS panels [60], Commercial evidence-based panels [57,61].

The ecological advantage of these panels lies in the “One Test, One Life” principle. By replacing hundreds of potential biochemical follow-up tests with a single genetic analysis, healthcare systems significantly reduce the consumption of single-use plastic goods, chemical reagents, energy, and transportation resources.

### 4.5. CYP-Based PGx as a Sustainable Alternative

#### 4.5.1. One Test, Lifetime Utility

The “one test, lifetime utility” principle represents a paradigm shift in diagnostic sustainability. Unlike traditional clinical biochemical monitoring following initiation of standard-dose therapy (e.g., therapeutic drug monitoring or liver function tests), which requires longitudinal sampling and repeated laboratory processing, an individual’s genomic profile remains constant throughout their lifetime [7]. A single, comprehensive multigene PGx panel provides a consistent reference for dozens of potential drug classes. This once-for-all approach significantly reduces the cumulative laboratory footprint—including the consumption of chemical reagents and single-use plastic consumables, and the energy-intensive logistics of repeated sample transport—and is inherently a greener alternative to continuous clinical monitoring. However, it should be remembered that the genetic primary data obtained once and for all are modified throughout life by acquired epigenetic patterns, and in many cases, phenotypic activity different from that genetically indicated (i.e., phenoconversion) is observed, indicating a metabolic activity different from that expected [62,63,64,65].

- Precision dosing is a critical strategy to reduce the overall chemical burden on the patient and the ecosystem. Traditional one-size-fits-all dosing often leads to excessive drug delivery in individuals who, due to their genetic makeup (e.g., IM or PM), require significantly lower doses to achieve therapeutic efficacy [2,66]. By tailoring the initial dose to the patient’s specific metabolic capacity, PGx avoids systemic drug overdose. This optimization ensures that only the minimum amount required for efficacy is delivered to the patient, thereby reducing the burden on the body.
- The environmental importance of CYP-mediated metabolism is a key part of this framework. CYP-catalyzed molecular oxygenation is a crucial first step in the environmental breakdown of synthetic xenobiotics. When a drug is administered to an individual with a PM phenotype, the phase I. oxidative biotransformation pathway is bypassed, leading to excretion of the persistent, unmetabolized parent compound rather than a more polar, biodegradable metabolite.

#### 4.5.2. Long-Term Resource Allocation

The financial and operational benefits of PGx—specifically regarding cost-effectiveness and the reduction of healthcare burdens—are increasingly evidenced by large-scale, real-world implementation programs [14]. A landmark application within the Kentucky Medicare Advantage program, which focused on older adults with a high burden of polypharmacy, provides robust longitudinal data [67]. An analysis of 5843 enrollees revealed that PGx insights prompted medication adjustments for nearly 70% of participants whose previous regimens were inconsistent with their genetic profiles.

Clinical and Economic Impact: Evidence from PGx-based programs shows significant reductions in acute care utilization—specifically, a 15% reduction in hospitalizations and a 6.8% reduction in emergency department visits, resulting in a total savings of $37 million over 32 months. Within the Eco-gene framework, these clinical outcomes are direct indicators of environmental sustainability. Every avoided hospitalization reduces resource-intensive medical waste and energy-intensive interventions, which typically yield the highest Eco-scores.

The first-fit model demonstrates that genetic-based drug optimization can be a primary tool for reducing proactive drug prescribing. By minimizing medication switching and eliminating ineffective prescriptions, we directly reduce drug waste and the excretion of persistent parent compounds—both of which would otherwise cause ecological toxicity [68].

#### 4.5.3. Limitations of Ethnicity-Based Dosing

**Figure 2:**
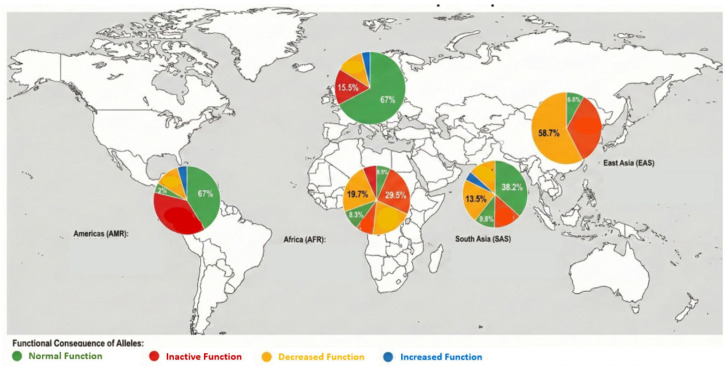
Global landscape of *CYP2D6* allelic distribution.

For decades, clinical pharmacology has relied on self-reported ethnicity as a rough proxy and a significantly simplified method for predicting drug response and metabolic capacity (pharmacokinetic and dynamic values) in mixed ancestry [69,70]. However, in the era of precision medicine, these traditional population models are increasingly recognized as scientifically inefficient and clinically risky. The human genome contains over 3 billion base pairs, and SNPs occur approximately every 1000 base pairs [6]. These variations account for nearly 90% of the genetic differences between individuals, profoundly influencing the expression and activity of drug-metabolizing enzymes (DMEs), transporters, and molecular targets [71]. Failure of ethnicity-based generalizations: Traditional models fail to account for significant individual variability within ethnic groups, antidepressant [70,72,73]. Not to mention the new, previously unknown combinations of unique variants that appear in descendants of different ethnicities. Although certain alleles show different geographical distributions, they are rarely exclusive to a single population: for example, *CYP2D6* alleles show significant frequency differences between continents (Figure 2); certain variants are significantly more common in African populations than in Asian or Caucasian cohorts [52,53]. Furthermore, significant enzymatic divergence at the proteomic level can also be observed. For example, *UGT1A1* polymorphisms associated with irinotecan toxicity are more common in East Asian populations, while *TPMT* variants affecting thiopurine metabolism are more common in Caucasian individuals [44,74]. Common drugs such as warfarin and 5-fluorouracil (5-FU) show highly variable responses based on these inherent genetic differences, making ethnicity-based dosing a game of trial and error rather than a precise medical intervention [69].

Data highlight significant inter-ethnic variability, including the high prevalence of the increased-function CYP2D6*10 allele in East Asian populations (58.7%) and the 4-splicing defect in Europeans (15.5%). These variations underscore the clinical necessity of the PGx-Green Passport over ethnicity-based dosing models.

### 4.6. The Challenge of Global Diversity and Genetic Admixture

The most compelling argument for individualized PGx is the genetic admixture of the 21st century [75]. Traditional medicine used ethnicity as a “rough proxy” for metabolism, but global population mixing has rendered this approach scientifically invalid.

As global migration and genetic admixture accelerate, traditional demographic proxies for drug prescribing have become obsolete. The human genome contains over 3 billion base pairs, with SNPs occurring approximately every 1000 base pairs [6]. These variations, which account for nearly 90% of human genetic differences, fundamentally alter the activity of drug-metabolizing enzymes, transporters, and receptors.

#### 4.6.1. The Hybridization Gap

In modern urban centers, patients often possess mixed genetic heritage (e.g., Euro-Asian or Afro-European). In these mixed individuals, metabolic activity cannot be inferred from physical appearance or self-reported ethnicity. Individual PGx testing is a potential universal safety standard that can predict drug response in a diverse, globalized society, ensuring that precision medicine is truly inclusive and environmentally friendly by preventing unnecessary or ineffective drug use and reducing ecotoxic waste.

In 2026, the concept of a genetically homogenous population is a relic of the past. Rapid global migration and increasing genetic admixture have created complex genomic mosaics [75]. In modern urban healthcare settings, a patient’s self-reported ethnicity often masks a hybridized genetic background that defies traditional dosing guidelines.

From a green pharmacy perspective, relying on these outdated surrogate methods is ecologically irresponsible. Therefore, moving beyond population-based models to individual PGx profiling is the only universal safety standard that ensures clinical efficacy while minimizing the systemic and environmental burden of modern pharmacotherapy [6,7]. It may sound paradoxical at this point, but individualized medication needs to be standardized.

#### 4.6.2. Molecular Mosaicism: The Impact of Compound Heterozygous Variants in Mixed Ancestry Populations

The limitations and errors of ethnicity-based drug dosing are increasingly apparent in the context of rapid global migration and genetic admixture, which introduce novel allelic variants, referred to as “molecular mosaicism,” within individual genomes (Figure 2). This issue is particularly pronounced at the *CYP2D6* locus, where traditional demographic distribution data fail to account for compound heterozygosity in individuals of mixed ethnicity. For instance, a child of mixed European and East Asian descent may inherit the nonfunctional CYP2D6*4 allele from the European parent, characterized by a G-to-A transition at rs3892097 (position 1846) that results in a severe splicing error [76,77]. Concurrently, the East Asian parent is statistically more likely to carry the reduced-function CYP2D6*10 allele, defined by a C-to-T transition at rs1065852 (position 100), leading to a Pro34Ser amino acid substitution [78,79]. The resulting offspring may possess a unique CYP2D6 *4/*10 genotype, exhibiting a complex molecular profile: heterozygous GA at position 1846 and heterozygous CT at position 100. Such individuals typically exhibit an IM phenotype with significantly reduced enzyme activity, a metabolic profile that cannot be accurately predicted from ethnicity or physical appearance. For mosaic genotypes with novel allelic combinations, reliance on universal dosing strategies is likely to result in clinical failure. For example, standard doses of codeine may lead to therapeutic failure, while standard doses of venlafaxine may cause toxicity. From an ecological perspective, this individual-level diagnostic inaccuracy due to demographic mixing is a significant driver of future pharmaceutical waste. Healthcare systems can mitigate the cycle of trial and error by utilizing PGx-based individual genomic data, such as the PGx-Green Passport, to guide the selection and dosing of biologically effective pharmaceuticals and minimize environmentally polluting pharmaceutical emissions. The molecular biological prerequisites for establishing these standard PGx-based databases are well understood.

#### 4.6.3. Genetic Admixture and Metabolic Unpredictability

To achieve truly eco-conscious medicine, we propose implementing a PGx-Green Passport. This digital, life-long genetic record follows the patient across borders and clinical settings, ensuring that every prescription is “first-time-right.”

Eco-Conscious Efficacy: By identifying ethnic-specific and individual genetic patterns pre-emptively, clinicians can avoid the trial-and-error loop that leads to unused or discarded medications [17,36,66,80].

Sustainability through Precision: The incorporation of PGx into clinical pipelines maximizes drug utility and minimizes healthcare expenditures by reducing the environmental burden of pharmaceutical waste.

Universal Applicability: Because inherited SNPs and other single-base variants remain constant throughout life, this “passport” serves as a constant reference, ensuring that drug therapy remains safe and effective regardless of a patient’s geographic location or ethnic background.

##### Bridging the Hybridization Gap

**Figure 3:**
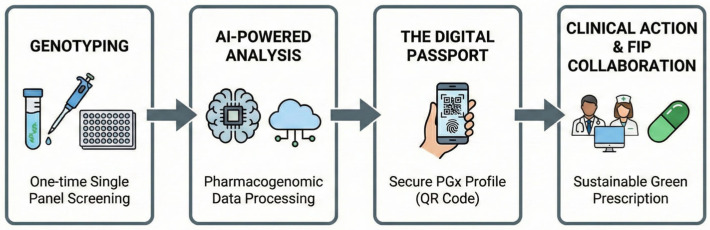
The PGx-Green Passport Workflow.

Amid increasing genetic admixture, the PGx-Green Passport overcomes the limitations of the unreliable one-size-fits-all approach based on demographic allele frequencies. It ensures patient treatment is tailored to the individual’s comprehensive genetic architecture, including SNPs, insertion-deletion (indel) variations, CNVs, and short tandem repeats (STRs). These genetic markers, especially indels and CNVs, are critical because they can affect mRNA stability or cause frameshift mutations, impacting protein expression and enzyme levels [23,64,81]. By focusing on the patient’s actual metabolic capacity rather than geographic origin, this approach aligns with the 3 PM healthcare model (prediction, prevention, and personalized medicine). It also supports a global strategy to reduce environmental and ecological footprints [63,64]. This Passport redefines PGx from a specialized clinical luxury to a fundamental, universal right to safe and sustainable healthcare (Figure 3).

### 4.7. The PGx-Green Passport: A Universal Safety Standard for a Rapidly Changing World

To address the inherent limitations of population- and ethnicity-based dosing and the growing environmental crisis, I propose introducing the PGx-Green Passport. While existing EHR integrations and CDS systems focus primarily on the patient-centric axis of drug safety and efficacy [22,60,61,62]. The PGx-Green Passport represents a paradigm shift by explicitly incorporating environmental accountability into the molecular-imprinting genetic registry. Unlike conventional CDS tools, which act as reactive alerts for individual toxicity, the Passport serves as a proactive, lifelong universal safety standard that remains clinically accurate and ecologically responsible across geographic and ethnic boundaries (Table 2).

The unique feature of the PGx-Green Passport is its systematic integration of Eco-scores directly into the therapeutic recommendation engine. Traditional PGx systems base dose adjustments on phenotypic predictions; however, the PGx-Green Passport enhances this by linking genetic variants to specific ecological outcomes. For example, neuroactive drugs such as SSRIs are regulated by the highly polymorphic CYP2D6 enzyme. Genotype-driven over-secretion alters chemical signals in the water. By reducing fluoxetine over-secretion through PGx-optimized dosing, the system lessens the disruption of chemical signals in wastewater—a direct environmental benefit that current clinical frameworks completely overlook.

Furthermore, the PGx-Green Passport addresses the resource efficiency gap inherent in traditional laboratory monitoring. Standard evidence-based models rely on repeated biochemical measurements, which entail continuous inventory logistics of reagents, plastic consumables, and energy-intensive supplies. In contrast, the Passport utilizes a single, comprehensive multigene panel that acts as a permanent reference for CYP enzymes, transporters (ABC, MDR, SLC), and drug targets [57]. This method not only avoids the “trial-and-error” disposal of ineffective drugs but also provides a high-level environmental safeguard at the point of care [19,56].

Unlike traditional concepts, the Passport does not just store raw genomic data. It also assigns integrated Eco-scores and evidence- or model-based ecological outcomes, such as bioaccumulation factors or endocrine disruption potential. Although the underlying pharmacogenetic network is highly complex, a CDS system manages this complexity. As a result, clinicians do not need to manually interpret ecotoxicological data. Instead, the PGx Green Passport offers a simplified, ecologically applicable recommendation that bridges molecular genetics and environmental awareness.

To explicitly illustrate the conceptual shift, Table 2 provides a focused contrast between traditional clinical decision support frameworks and the multi-layered environmental accountability of the PGx-Green Passport.

### 4.8. Global Policy Frameworks and the Evolution of Green Pharmacy

The integration of PGx into sustainable healthcare is not merely a clinical opportunity but a response to a global mandate. In 2016, the International Pharmaceutical Federation (FIP) published a landmark policy statement on Green Pharmacy Practice, formally acknowledging the environmental impact of medicines as a challenge of global significance [56,82]. The concept of Green Pharmacy has emerged as a necessary response to the growing body of evidence on the harmful environmental effects of pharmaceutical substances. It encompasses a holistic suite of measures designed to minimize the environmental footprint of medicines throughout their entire life cycle: Designing molecules with lower ecotoxicity, ensuring that every drug dispensed is biologically appropriate for the patient, a goal directly served by PGx-guided therapy, and reducing the volume of unconsumed medications that enter the waste stream. The FIP policy emphasizes that pharmacists must collaborate with prescribers to raise awareness of medicines’ environmental classifications and promote rational prescribing. We argue that in the era of precision medicine, rational prescribing is no longer possible without considering the patient’s genetic capacity to metabolize the drug. By incorporating environmental impact advice and PGx-based decision support (e.g., Eco-scores), the pharmacist-prescriber team can ensure that only efficacious, non-toxic doses are used, thereby fulfilling the FIP mandate for sustainable practice (Figure 3) [82]. The complex nature of multi-layered genomic data, which includes SNPs, insertions and deletions (indels), and structural variations, alongside clinical parameters, demands AI and machine learning to achieve the precision necessary for sustainable drug prescribing [83].

The conceptual framework of the PGx-Green Passport illustrates a closed-loop system that aligns clinical excellence with environmental stewardship.

Step 1—Genotyping: A comprehensive, one-time multigene panel identifies key CYP polymorphisms (*CYP2D6*, *CYP2C19*, *CYP2C9*, etc.), offering a resource-efficient “once-per-lifetime” diagnostic event that minimizes laboratory waste. Step 2—AI-Powered Analysis: Raw genomic data are processed through advanced machine learning algorithms to interpret complex metabolic phenotypes. This stage eliminates the cognitive load on healthcare providers and translates high-density SNP data into actionable clinical insights. Step 3—The Digital Passport: The interpreted data are stored in a portable, lifelong digital interface. This digital ID bypasses unreliable ethnicity-based proxies, providing a universal safety standard for a globalized, hybridized population. Step 4—Clinical Action and FIP Collaboration: In accordance with FIP guidelines, pharmacists and prescribers utilize the Passport to ensure “first-time-right” prescriptions. This collaborative action directly reduces the environmental burden by preventing ADRs and eliminating the disposal of ineffective medications into the ecosystem.

### 4.9. Technological and Educational Barriers to Implementation

Despite the clear clinical and ecological benefits of PGx, several systemic barriers hinder its widespread integration into routine practice. One major technological barrier is the lack of standardized CDS systems; without these digital tools, it remains difficult for clinicians to translate vast amounts of raw genomic data into practical, real-time decisions on drug type and optimal dose [28,84]. In addition, significant knowledge gaps exist among healthcare providers. Many physicians and pharmacists feel underprepared to interpret complex CYP genotypes and metabolic profiles, or to counsel patients at the intersection of genetic information and rational standard dose principles, a collaboration that FIP specifically encourages. In this context, AI serves as a critical catalyst for sustainable PGx. AI and machine learning algorithms are uniquely able to process the multi-layered complexity of multi-gene panels, not only predicting the risk of adverse events but also identifying opportunities for green interventions, such as proactive prescribing reductions to minimize drug waste [45,71,85]. However, increased reliance on high-performance computing imposes significant environmental burdens, mainly due to higher energy consumption and the carbon footprint of data centers [40,86,87]. Therefore, it is vital to deploy AI ethically and thoughtfully, ensuring computational resources are allocated to sectors with the highest social and ecological benefits.

Within the “One Health” framework, which links human health to ecosystem health, leveraging AI to optimize PGx-driven therapies is essential. Until human analytical capabilities or more energy-efficient computational models develop enough to reduce pharmaceutical environmental contamination, AI remains a necessary short-term solution. By accelerating the transition to accurate, eco-friendly applications, the energy costs of AI are offset by a notable reduction in long-term ecological harm from pharmaceutical pollutants, safeguarding the health of the planet and its inhabitants.

## 5. Discussion and Future Perspectives

The findings of this review demonstrate that integrating PGx into routine clinical practice is no longer a localized healthcare decision but a global ecological necessity. By bridging the gap between molecular biology and environmental stewardship, we move beyond the outdated “one-size-fits-all” model toward a sustainable, high-precision healthcare ecosystem where genetic data serves as a predictive tool for both clinical and environmental outcomes.

### 5.1. The Convergence of Clinical Excellence and Ecological Ethics

Our analysis confirms that clinical efficacy and environmental sustainability are fundamentally linked. The traditional trial-and-error prescribing model is a primary driver of pharmaceutical waste, resulting in the discharge of unused medications and ecotoxic metabolites into global wastewater systems. This synergy is now operationalized through the Eco-gene Matrix and the Eco-score system, which allows for the quantitative assessment of environmental risk alongside clinical safety.

The implementation of CYP-based individualized treatment protocols addresses this at the source:

- Waste Mitigation: By ensuring “first-time-right” prescribing, PGx reduces the systemic volume of unused drugs, fulfilling the FIP 2016 mandate for Green Pharmacy [6,82]. This directly prevents “non-responder waste,” where a 100% environmental burden is generated without any therapeutic gain.
- Resource Optimization: As evidenced by the Kentucky Medicare Advantage program, PGx-informed interventions can reduce hospitalizations by 15% and generate over $37 million in savings, proving that economic viability and patient safety are synergistic [68]. Within a sustainability framework, these savings represent a massive reduction in the high-resource medical waste and energy-intensive logistics associated with acute clinical failures.

### 5.2. The PGx-Green Passport: Navigating Genetic Admixture

As global populations become increasingly hybridized, relying on ethnic or geographic proxies for drug metabolism is scientifically flawed and clinically dangerous. The human genome’s SNP density (1 per 1000 bases) creates metabolic diversity that transcends traditional categories [69].

The proposed PGx-Green Passport serves as a universal safety standard, providing a digital, lifelong genetic record that ensures medication safety regardless of a patient’s mixed heritage. The proposed PGx-Green Passport transcends conventional EHR integrations and CDS tools, which remain largely reactive and patient-centric.

Unlike standard CDS frameworks, the PGx-Green Passport functions as a proactive, lifelong molecular registry that explicitly accounts for ecological ADRs. By integrating Eco-scores directly into the therapeutic recommendation engine, the Passport alerts clinicians when a patient’s genotype (e.g., PM) may result in the excretion of persistent, ecotoxic parent compounds. This approach transforms the 3 PM healthcare model into a tangible reality for a globalized society [88].

### 5.3. A Pragmatic Approach to Green Pharmacy

Engineering inherent biodegradability into drug molecules remains an immense challenge in medicinal chemistry, as structural modifications intended to facilitate environmental breakdown frequently result in detrimental pharmacodynamic alterations and a loss of therapeutic efficacy. Furthermore, it must be emphasized that CYP-catalysed molecular oxygenation is the essential first step in facilitating the environmental degradation of most synthetic xenobiotics. Recognizing these limitations, the proposed Green PGx framework approaches environmental sustainability not through molecular redesign, but by optimizing clinical utility to minimize the administration of ineffective pharmacons. Patients with a PM phenotype represent a critical ecological risk group; due to impaired oxidative pathways, they excrete persistent, unmetabolized parent drugs rather than more polar, easily degradable metabolites. By ensuring that therapy is precisely matched to the patient’s metabolic capacity, this model directly reduces the environmental excretion of both persistent parent compounds and potentially ecotoxic metabolites originating from non-responsive treatments. In this context, Green PGx serves as a pragmatic bridge, where green chemistry meets its practical limits, transforming clinical efficiency into a primary tool for environmental protection.

## 6. Conclusions

In summary, PGx extends beyond clinical efficacy optimization; it represents a critical advancement in green pharmacy and is essential for sustainable medicine. Transitioning from a reactive, uniform prescribing model based on standard body weight or body surface area to a proactive PGx-Green Passport framework enables healthcare systems to maximize therapeutic efficacy, enhance patient safety, and substantially reduce the environmental impact of pharmacotherapy. Evidence shows that individual genetic profiling, especially for CYP enzyme activity, offers a dependable safety standard capable of managing the biological complexity of mixed genetic populations, where traditional demographic indicators are no longer sufficient. The use of multigene panels in routine clinical practice provides the benefits of reducing hospital visits and decreasing the systemic chemical load released into global ecosystems. Identifying PM phenotypes is now more than a clinical safety measure; it becomes a vital environmental safeguard to stop the release of persistent, unmetabolized drug compounds into global wastewater systems. The core innovation of this transition lies in integrating the Eco-gene Matrix and the Eco-score, which provides a quantitative methodology for linking human metabolic variability to specific ecological outcomes. The long-term success of this transition will require unprecedented interdisciplinary collaboration among clinicians, pharmacists, bioinformaticians, and policymakers. Adoption of the PGx-Green Passport as a universal baseline standard can ensure that precision medicine safeguards both human health and ecological integrity, preventing life-saving medicines from becoming persistent environmental pollutants.

## Figures and Tables

**Table 1:** The Eco-gene Matrix—Linking PGx Variants to Environmental Toxicity. This table categorizes risk by Eco-score (1–10), where 10 represents the highest environmental hazard (high potency, high persistence, and a significant increase in genotype-driven excretion). Abbreviation: PM: Poor Metabolizer; IM: Intermediate Metabolizer; EE2: 17α-Ethinylestradiol; NSAID: Non-Steroidal Anti-Inflammatory Drugs; SSRI: Selective Serotonin Reuptake Inhibitors.

Gene/Variant	Clinical Phenotype	Target Compounds	Environmental Excretion Mechanism	Ecotoxicological Impact	Eco-Score
CYP3A4*22	PM/IM	Synthetic Steroids (EE2, Dexamethasone)	Increased parent compound density in urine	Endocrine disruption: Reproductive failure in fish; feminization of aquatic species.	10
CYP2C9*3	PM	NSAIDs (Diclofenac, Celecoxib)	Failure to oxidize; excretion of persistent parent drug.	Aquatic Toxicity: Renal/hepatic damage in vultures and fish; high persistence.	9
UGT1A1*28	Reduced Conjugation	Estrogens, Irinotecan	Shift from water-soluble conjugates to lipophilic forms.	Bioaccumulation: Higher bioaccumulation Factor in adipose tissues.	8
CYP2D6*4/5	Loss of Function (PM)	SSRIs (Fluoxetine), Beta-blockers	Higher concentration of neuroactive molecules in wastewater.	Behavioral Ecotox: Altered predator-prey response and migration patterns.	7
*ABCB1 3435T*	Altered Transport	Corticosteroids, Digoxin	Altered ratio between renal (urine) and biliary (fecal) excretion.	Environmental Fate: Challenges in wastewater filtration efficiency.	6

**Table 2:** Functional Contrast: CDS vs. the Proposed PGx-Green Passport.

Feature	Conventional CDS	Proposed PGx-Green Passport
Primary Focus	Patient safety and clinical efficacy.	Patient safety integrated with environmental sustainability.
Core Metric	Pharmacokinetic status (e.g., PM/UM).	Integrated Eco-Score (Metabolism + Persistence + Potency).
Risk Driving Factor	ADRs in patients.	Ecological ADRs in non-target species and ecosystems.
Operational Logic	Reactive: Point-of-care clinical alerts.	Proactive: Life-long metabolic and ecological blueprint.
Resource Impact	Clinical cost and hospital stay reduction.	Source-reduction of pharmaceutical and medical waste.
Population Scope	Demographic and ethnic statistical proxies.	Individual genetic architecture (Admixture-ready).

## Data Availability

No new data were created or analyzed in this study. Data sharing is not applicable to this article.

## Abbreviations

ADR	Adverse Drug Reaction
AI	Artificial Intelligence
API	Active Pharmaceutical Ingredient
CDS	Clinical Decision Support
CPIC	Clinical Pharmacogenetics Implementation Consortium
CYP	Cytochrome P450 enzyme
DPWG	Dutch Pharmacogenetics Working Group
EHR	Electronic Health Record
FIP	International Pharmaceutical Federation
IM	Intermediate Metabolizer
NGS	Next-Generation Sequencing
NSAID	Non-Steroidal Anti-Inflammatory Drugs
PGx	Pharmacogenomics
PM	Poor Metabolizer
SNP	Single Nucleotide Polymorphism
SSRI	Selective Serotonin Reuptake Inhibitors
STR	Short Tandem Repeats
UM	Ultrarapid Metabolizer
WHO	World Health Organization
WWTP	Wastewater Treatment Plant

## References

[1] Sissung T.M., McKeeby J.W., Patel J., Lertora J.J., Kumar P., Flegel W.A., Adams S.D., Eckes E.J., Mickey F., Plona T.M. (2017). Pharmacogenomics Implementation at the National Institutes of Health Clinical Center. J. Clin. Pharmacol..

[2] Hughes C.M. (2022). One Size Fits All? How to Optimize the Prescribing of Appropriate Polypharmacy in Chronic Diseases, Using a Behavioral Approach—A United Kingdom Perspective. Expert Rev. Clin. Pharmacol..

[3] Schwaederle M., Zhao M., Lee J.J., Eggermont A.M., Schilsky R.L., Mendelsohn J., Lazar V., Kurzrock R. (2015). Impact of Precision Medicine in Diverse Cancers: A Meta-Analysis of Phase II Clinical Trials. J. Clin. Oncol..

[4] Caudle K.E., Klein T.E., Hoffman J.M., Muller D.J., Whirl-Carrillo M., Gong L., McDonagh E.M., Sangkuhl K., Thorn C.F., Schwab M. (2014). Incorporation of Pharmacogenomics into Routine Clinical Practice: The Clinical Pharmacogenetics Implementation Consortium (CPIC) Guideline Development Process. Curr. Drug Metab..

[5] Roden D.M., McLeod H.L., Relling M.V., Williams M.S., Mensah G.A., Peterson J.F., Van Driest S.L. (2019). Pharmacogenomics. Lancet.

[6] Lakshmi K., Nagarajan B., Dabburu K., Roy C., Shanthi B., Das G., Chakraborty T., Syed S., Albert J., Prabha K.S. (2026). Pharmacogenomics for Sustainable Drug Development: A Narrative Review of Precision Medicine, Green Chemistry, and Multi-Omics Innovation. J. Appl. Pharm. Sci..

[7] Relling M.V., Evans W.E. (2015). Pharmacogenomics in the Clinic. Nature.

[8] Swen J.J., Nijenhuis M., de Boer A., Grandia L., Maitland-van der Zee A.H., Mulder H., Rongen G.A.P.J.M., van Schaik R.H.N., Schalekamp T., Touw D.J. (2011). Pharmacogenetics: From Bench to Byte—An Update of Guidelines. Clin. Pharmacol. Ther..

[9] Hicks J.K., Bishop J.R., Sangkuhl K., Müller D.J., Ji Y., Leckband S.G., Leeder J.S., Graham R.L., Chiulli D.L., LLerena A. (2015). Clinical Pharmacogenetics Implementation Consortium (CPIC) Guideline for *CYP2D6* and *CYP2C19* Genotypes and Dosing of Selective Serotonin Reuptake Inhibitors. Clin. Pharmacol. Ther..

[10] Crews K.R., Gaedigk A., Dunnenberger H.M., Leeder J.S., Klein T.E., Caudle K.E., Haidar C.E., Shen D.D., Callaghan J.T., Sadhasivam S. (2014). Clinical Pharmacogenetics Implementation Consortium guidelines for cytochrome P450 2D6 genotype and codeine therapy: 2014 update. Clin. Pharmacol. Ther..

[11] Lima J.J., Thomas C.D., Barbarino J., Desta Z., Van Driest S.L., El Rouby N., Johnson J.A., Cavallari L.H., Shakhnovich V., Thacker D.L. (2020). Clinical Pharmacogenetics Implementation Consortium (CPIC) Guideline for *CYP2C19* and Proton Pump Inhibitor Dosing. Clin. Pharmacol. Ther..

[12] Bousman C.A., Stevenson J.M., Ramsey L.B., Sangkuhl K., Hicks J.K., Strawn J.R., Singh A.B., Ruaño G., Mueller D.J., Tsermpini E.E. (2023). Clinical Pharmacogenetics Implementation Consortium (CPIC) Guideline for *CYP2D6*, *CYP2C19*, *CYP2B6*, *SLC6A4*, and *HTR2A* Genotypes and Serotonin Reuptake Inhibitor Antidepressants. Clin. Pharmacol. Ther..

[13] Baum M.L., Widge A.S., Carpenter L.L., McDonald W.M., Cohen B.M., Nemeroff C.B., On behalf of the American Psychiatric Association (APA) Workgroup on Biomarkers Novel (2024). Pharmacogenomic Clinical Support Tools for the Treatment of Depression. Am. J. Psychiatry.

[14] Jiang S., Mathias P.C., Hendrix N., Shirts B.H., Tarczy-Hornoch P., Veenstra D., Malone D., Devine B. (2022). Implementation of Pharmacogenomic Clinical Decision Support for Health Systems: A Cost-Utility Analysis. Pharmacogenomics J..

[15] van der Wouden C.H., Cambon-Thomsen A., Cecchin E., Cheung K.C., Dávila-Fajardo C.L., Deneer V.H., Dolžan V., Ingelman-Sundberg M., Jönsson S., Karlsson M.O. (2017). Implementing Pharmacogenomics in Europe: Design and Implementation Strategy of the Ubiquitous Pharmacogenomics Consortium. Clin. Pharmacol. Ther..

[16] Phillips K.A., Veenstra D.L., Oren E., Lee J.K., Sadee W. (2001). Potential Role of Pharmacogenomics in Reducing Adverse Drug Reactions: A Systematic Review. JAMA.

[17] Caudle K.E., Dunnenberger H.M., Freimuth R.R., Peterson J.F., Burlison J.D., Whirl-Carrillo M., Scott S.A., Rehm H.L., Williams M.S., Klein T.E. (2017). Standardizing Terms for Clinical Pharmacogenetic Test Results: Guidance from the Clinical Pharmacogenetics Implementation Consortium (CPIC). Genet. Med..

[18] Daughton C.G., Ruhoy I.S. (2010). Reducing the Ecological Footprint of Pharmaceutical Usage: Linkages Between Healthcare Practices and the Environment. Green and Sustainable Pharmacy.

[19] Salei E., Persson C.L., Håkonsen H. (2025). Green Pharmacy Practice—A Multi Method Study of Environmental Sustainability Measures Implemented in Swedish Pharmacies. J. Pharm. Policy Pract..

[20] Khetan S.K., Collins T.J. (2007). Human Pharmaceuticals in the Aquatic Environment: A Challenge to Green Chemistry. Chem. Rev..

[21] Relling M.V., Klein T.E. (2011). CPIC: Clinical Pharmacogenetics Implementation Consortium of the Pharmacogenomics Research Network. Clin. Pharmacol. Ther..

[22] Daughton C.G. (2003). Cradle-to-Cradle Stewardship of Drugs for Minimizing Their Environmental Disposition While Promoting Human Health. II. Drug Disposal, Waste Reduction, and Future Directions. Environ. Health Perspect..

[23] Tibben B.M., Gaedigk A., Gong L., Sangkuhl K., Whirl-Carrillo M., Relling M.V., Donnelly R.S., Klein T.E., Caudle K.E. (2025). The Clinical Pharmacogenetics Implementation Consortium’s Consensus-Based Framework for Assigning Allele Function. Am. J. Hum. Genet..

[24] Environmentally Sustainable Health Systems: A Strategic Document. https://www.who.int/publications/i/item/WHO-EURO-2017-2241-41996-57723.

[25] Wheeler H.E., Maitland M.L., Dolan M.E., Cox N.J., Ratain M.J. (2013). Cancer Pharmacogenomics: Strategies and Challenges. Nat. Rev. Genet..

[26] Evans W.E., McLeod H.L. (2003). Pharmacogenomics—Drug Disposition, Drug Targets, and Side Effects. N. Engl. J. Med..

[27] Alshemari A., Breen L., Quinn G., Sivarajah U. (2020). Can We Create a Circular Pharmaceutical Supply Chain (CPSC) to Reduce Medicines Waste?. Pharmacy.

[28] Dunnenberger H.M., Crews K., Hoffman J., Caudle K., Broeckel U., Howard S., Hunkler R., Klein T., Evans W., Relling M. (2014). Preemptive Clinical Pharmacogenetics Implementation: Current Programs in Five US Medical Centers. Annu. Rev. Pharmacol. Toxicol..

[29] Whirl-Carrillo M., McDonagh E., Hebert J., Gong L., Sangkuhl K., Thorn C., Altman R., Klein T. (2012). Pharmacogenomics Knowledge for Personalized Medicine. Clin. Pharmacol. Ther..

[30] van der Wouden C.H., van Rhenen M.H., Jama W.O.M., Ingelman-Sundberg M., Lauschke V.M., Konta L., Schwab M., Swen J.J., Guchelaar H.-J. (2019). Development of the PGx-Passport: A Panel of Actionable Germline Genetic Variants for Pre-Emptive Pharmacogenetic Testing. Clin. Pharmacol. Ther..

[31] Hamzic S., Aebi S., Joerger M., Montemurro M., Ansari M., Amstutz U., Largiadr C. (2020). Fluoropyrimidine Chemotherapy: Recommendations for DPYD Genotyping and Therapeutic Drug Monitoring of the Swiss Group of Pharmacogenomics and Personalised Therapy. Swiss Med. Wkly..

[32] Dunnenberger H.M., Biszewski M., Bell G.C., Sereika A., May H., Johnson S.G., Hulick P.J., Khandekar J. (2016). Implementation of a multidisciplinary pharmacogenomics clinic in a community health system. Am. J. Health Syst. Pharm..

[33] Lauschke V.M., Ingelman-Sundberg M. (2016). Precision Medicine and Rare Genetic Variants. Trends Pharmacol. Sci..

[34] Zinken J.F., Pasmooij A.M.G., Ederveen A.G.H., Hoekman J., Bloem L.T. (2024). Environmental Risk Assessment in the EU Regulation of Medicines for Human Use: An Analysis of Stakeholder Perspectives on Its Current and Future Role. Drug Discov. Today.

[35] Lattanzio S., Stefanizzi P., D’ambrosio M., Cuscianna E., Riformato G., Migliore G., Tafuri S., Bianchi F.P. (2022). Waste Management and the Perspective of a Green Hospital—A Systematic Narrative Review. Int. J. Environ. Res. Public Health.

[36] Evans W.E., Relling M.V. (2004). Moving towards Individualized Medicine with Pharmacogenomics. Nature.

[37] Mosch R., van der Lee M., Guchelaar H.J., Swen J.J. (2025). Pharmacogenetic Panel Testing: A Review of Current Practice and Potential for Clinical Implementation. Annu. Rev. Pharmacol. Toxicol..

[38] Jeyavani J., Sibiya A., Stalin T., Vigneshkumar G., Al-Ghanim K.A., Riaz M.N., Govindarajan M., Vaseeharan B. (2023). Biochemical, Genotoxic and Histological Implications of Polypropylene Microplastics on Freshwater Fish Oreochromis Mossambicus: An Aquatic Eco-Toxicological Assessment. Toxics.

[39] Occurrence, Ecotoxicological Effects and Risk Assessment of Antihypertensive Pharmaceutical Residues in the Aquatic Environment—A Review. https://www.researchgate.net/publication/278676558_Occurrence_ecotoxicological_effects_and_risk_assessment_of_antihypertensive_pharmaceutical_residues_in_the_aquatic_environment_-_A_review.

[40] Samuel G., Lucassen A.M. (2022). The Environmental Impact of Data-Driven Precision Medicine Initiatives. Camb. Prism. Precis. Med..

[41] Wilkinson G.R. (2005). Drug Metabolism and Variability among Patients in Drug Response. N. Engl. J. Med..

[42] Reeve J., Maden M., Hill R., Turk A., Mahtani K., Wong G., Lasserson D., Krska J., Mangin D., Byng R. (2022). Deprescribing Medicines in Older People Living with Multimorbidity and Polypharmacy: The TAILOR Evidence Synthesis. Health Technol. Assess. Winch. Engl..

[43] Zhou S.-F., Liu J.-P., Chowbay B. (2009). Polymorphism of Human Cytochrome P450 Enzymes and Its Clinical Impact. Drug Metab. Rev..

[44] Pirmohamed M., Park B.K. (2001). Genetic Susceptibility to Adverse Drug Reactions. Trends Pharmacol. Sci..

[45] Onder G., Lattanzio F., Battaglia M., Cerullo F., Sportiello R., Bernabei R., Landi F. (2011). The Risk of Adverse Drug Reactions in Older Patients: Beyond Drug Metabolism. Curr. Drug Metab..

[46] Swen J.J., van der Wouden C.H., Manson L.E., Abdullah-Koolmees H., Blagec K., Blagus T., Böhringer S., Cambon-Thomsen A., Cecchin E., Cheung K.-C. (2023). A 12-Gene Pharmacogenetic Panel to Prevent Adverse Drug Reactions: An Open-Label, Multicentre, Controlled, Cluster-Randomised Crossover Implementation Study. Lancet.

[47] Scott S.A., Sangkuhl K., Stein C.M., Hulot J.-S., Mega J.L., Roden D.M., Klein T.E., Sabatine M.S., Johnson J.A., Shuldiner A.R. (2013). Clinical Pharmacogenetics Implementation Consortium Guidelines for *CYP2C19* Genotype and Clopidogrel Therapy: 2013 Update. Clin. Pharmacol. Ther..

[48] Cavallari L.H., Lee C.R., Beitelshees A.L., Cooper-DeHoff R.M., Duarte J.D., Voora D., Kimmel S.E., McDonough C.W., Gong Y., Dave C.V. (2018). Multi-Site Investigation of Outcomes with Implementation of *CYP2C19* Genotype-Guided Antiplatelet Therapy after Percutaneous Coronary Intervention. JACC Cardiovasc. Interv..

[49] Zhou Y., Nevosadová L., Eliasson E., Lauschke V.M. (2023). Global Distribution of Functionally Important CYP2C9 Alleles and Their Inferred Metabolic Consequences. Hum. Genom..

[50] Desta Z., Gammal R.S., Gong L., Whirl-Carrillo M., Gaur A.H., Sukasem C., Hockings J., Myers A., Swart M., Tyndale R.F. (2019). Clinical Pharmacogenetics Implementation Consortium (CPIC) Guideline for CYP2B6 and Efavirenz-Containing Antiretroviral Therapy. Clin. Pharmacol. Ther..

[51] Brixner D., Biltaji E., Bress A., Unni S., Ye X., Mamiya T., Ashcraft K., Biskupiak J. (2016). The Effect of Pharmacogenetic Profiling with a Clinical Decision Support Tool on Healthcare Resource Utilization and Estimated Costs in the Elderly Exposed to Polypharmacy. J. Med. Econ..

[52] Peters B.J.M., Klungel O.H., de Boer A., Ch Stricker B.H., Maitland-van der Zee A.-H. (2009). Pharmacogenetics of Cardiovascular Drug Therapy. Clin. Cases Miner. Bone Metab..

[53] Challenges in Clinical Implementation of *CYP2D6* Genotyping: Choice of Variants to Test Affects Phenotype Determination—Genetics in Medicine. https://www.gimjournal.org/article/S1098-3600(21)01108-4/fulltext.

[54] Zhang L., Tholkes A.J., Jones K.C., Yang L.J., Sieger G.K., Cullen K.R., Gunlicks-Stoessel M.L., Mroz P., Farley J.F., Johnson S.G. (2025). Real-World Characterization of Psychiatric Pharmacogenomic Test Ordering and Clinical Relevance in Adults and Children. Clin. Transl. Sci..

[55] Molla G., Bitew M. (2024). Revolutionizing Personalized Medicine: Synergy with Multi-Omics Data Generation, Main Hurdles, and Future Perspectives. Biomedicines.

[56] Daughton C., Ruhoy I. (2011). Green Pharmacy and pharmEcovigilance: Prescribing and the Planet. Expert Rev. Clin. Pharmacol..

[57] El Rouby N., Allen J.D., Koep T., McIntyre P., Kelly J., Ramesh A., Desai-Naik A., Chiang J., Patel S., Brueckner C. (2025). Multi-Gene Pharmacogenomic Testing in a Community-Based Setting Is Feasible and Reduces Total Healthcare Costs. Clin. Transl. Sci..

[58] Table of Pharmacogenomic Biomarkers in Drug Labeling. https://www.fda.gov/drugs/science-and-research-drugs/table-pharmacogenomic-biomarkers-drug-labeling.

[59] de Leon J., Susce M.T., Murray-Carmichael E. (2006). The AmpliChip *CYP450* Genotyping Test: Integrating a New Clinical Tool. Mol. Diagn. Ther..

[60] Barbarino J.M., Whirl-Carrillo M., Altman R.B., Klein T.E. (2018). PharmGKB: A Worldwide Resource for Pharmacogenomic Information. Wiley Interdiscip. Rev. Syst. Biol. Med..

[61] GeneSight Testing: Your DNA Holds the Key to Finding the Right Antidepressant. https://www.reviveholisticpsychiatry.com/post/genesight-testing-your-dna-holds-the-key-to-finding-the-right-antidepressant.

[62] Klomp S.D., Manson M.L., Guchelaar H.-J., Swen J.J. (2020). Phenoconversion of Cytochrome P450 Metabolism: A Systematic Review. J. Clin. Med..

[63] Michaud V., Dow P., Turgeon J. (2021). Illustrative and historic cases of phenoconversion. Am. J. Transl. Res..

[64] Auwerx C., Sadler M.C., Reymond A., Kutalik Z. (2022). From Pharmacogenetics to Pharmaco-Omics: Milestones and Future Directions. Hum. Genet. Genom. Adv..

[65] de Jong L.M., Boussallami S., Sánchez-López E., Giera M., Tushuizen M.E., Hoekstra M., Hawinkels L.J.A.C., Rissmann R., Swen J.J., Manson M.L. (2023). The Impact of *CYP2C19* Genotype on Phenoconversion by Concomitant Medication. Front. Pharmacol..

[66] Turner R.M., Park B.K., Pirmohamed M. (2015). Parsing Interindividual Drug Variability: An Emerging Role for Systems Pharmacology. Wiley Interdiscip. Rev. Syst. Biol. Med..

[67] Real-World Impact of a Pharmacogenomics-Enriched Comprehensive Medication Management Program. https://www.mdpi.com/2075-4426/12/3/421.

[68] Pharmaceutical Waste Management: A Comprehensive Analysis of Romanian Practices and Perspectives. https://www.mdpi.com/2071-1050/16/15/6571.

[69] Ortega V.E., Meyers D.A. (2014). Pharmacogenetics: Implications of Race and Ethnicity on Defining Genetic Profiles for Personalized Medicine. J. Allergy Clin. Immunol..

[70] Popejoy A.B., Fullerton S.M. (2016). Genomics Is Failing on Diversity. Nature.

[71] Lettieri M., Rodda M., Carlucci V. (2025). Machine Learning in Early Prediction of Metabolism of Drugs. Methods Mol. Biol..

[72] Zhou Y., Ingelman-Sundberg M., Lauschke V. (2017). Worldwide Distribution of Cytochrome P450 Alleles: A Meta-analysis of Population-scale Sequencing Projects. Clin. Pharmacol. Ther..

[73] Narasimhan S., Lohoff F.W. (2012). Pharmacogenetics of antidepressant drugs: Current clinical practice and future directions. Pharmacogenomics.

[74] McLeod H.L., Krynetski E.Y., Relling M.V., Evans W.E. (2000). Genetic Polymorphism of Thiopurine Methyltransferase and Its Clinical Relevance for Childhood Acute Lymphoblastic Leukemia. Leukemia.

[75] Esperón P., Martínez M.F., Redal M.A., Lazarowski A., López-Cortés A., Varela N.M., Quiñones L.A. (2022). Editorial: Pharmacogenetics and Pharmacogenomics in Latin America: Ethnic Variability, New Insights in Advances and Perspectives: A RELIVAF-CYTED Initiative. Front. Pharmacol..

[76] Salyakina D., Roy S., Wang W., Oliva M., Akhouri R., Sotto I., Mulas N., Solano R., Fernandez J., Sanchez S. (2019). Results and Challenges of Cytochrome P450 2D6 (*CYP2D6*) Testing in an Ethnically Diverse South Florida Population. Mol. Genet. Genom. Med..

[77] Anwarullah, Aslam M., Badshah M., Abbasi R., Sultan A., Khan K., Ahmad N., von Engelhardt J. (2017). Further Evidence for the Association of *CYP2D6**4 Gene Polymorphism with Parkinson’s Disease: A Case Control Study. Genes Environ..

[78] Wei H., Zhao Q. (2025). *CYP2D6* Polymorphism Rs1065852 Significantly Increases the Risk of Type 2 Diabetes. Ann. Med..

[79] Bagheri A., Kamalidehghan B., Haghshenas M., Azadfar P., Akbari L., Sangtarash M.H., Vejdandoust F., Ahmadipour F., Meng G.Y., Houshmand M. (2015). Prevalence of the CYP2D6*10 (C100T), *4 (G1846A), and *14 (G1758A) alleles among Iranians of different ethnicities. Drug Des. Dev. Ther..

[80] van der Wouden C.H., Marck H., Guchelaar H.-J., Swen J.J., van den Hout W.B. (2022). Cost-Effectiveness of Pharmacogenomics-Guided Prescribing to Prevent Gene-Drug-Related Deaths: A Decision-Analytic Model. Front. Pharmacol..

[81] Magliocco G., Desmeules J., Matthey A., Quirós-Guerrero L., Bararpour N., Joye T., Marcourt L., Queiroz E., Wolfender J.-L., Gloor Y. (2021). Metabolomics Reveals Biomarkers in Human Urine and Plasma to Predict Cytochrome P450 2D6 (*CYP2D6*) Activity. Br. J. Pharmacol..

[82] Fip Statement of Policy: Environmental Sustainability within Pharmacy. https://www.researchgate.net/publication/377599848_FIP_STATEMENT_OF_POLICY_Environmental_sustainability_within_pharmacy.

[83] Sonaji P., Subramanian L., Rajesh M. (2024). Artificial Intelligence-Driven Drug Interaction Prediction. World J. Biol. Pharm. Health Sci..

[84] Crews K.R., Cross S.J., McCormick J.N., Baker D.K., Molinelli A.R., Mullins R., Relling M.V., Hoffman J.M. (2011). Development and Implementation of a Pharmacist-Managed Clinical Pharmacogenetics Service. Am. J. Health-Syst. Pharm. AJHP Off. J. Am. Soc. Health-Syst. Pharm..

[85] Johnson K.B., Wei W.-Q., Weeraratne D., Frisse M.E., Misulis K., Rhee K., Zhao J., Snowdon J.L. (2021). Precision Medicine, AI, and the Future of Personalized Health Care. Clin. Transl. Sci..

[86] Samuel G., Lucassen A.M. (2022). The Environmental Sustainability of Data-Driven Health Research: A Scoping Review. Digit. Health.

[87] Griffiths J., Fox L., Williamson P.R. (2024). Quantifying the Carbon Footprint of Clinical Trials: Guidance Development and Case Studies. BMJ Open.

[88] The Fate of Medicines after Use and Their Role in Environmental Pollution—Pharmacy English Site 2026. https://pharmacy.uokerbala.edu.iq/wp/en/blog/2026/01/28/the-fate-of-medicines-after-use-and-their-role-in-environmental-pollution/.

